# Provincial Dietary Intake Study (PDIS): Micronutrient Intakes of Children in a Representative/Random Sample of 1- to <10-Year-Old Children in Two Economically Active and Urbanized Provinces in South Africa

**DOI:** 10.3390/ijerph17165924

**Published:** 2020-08-14

**Authors:** Marjanne Senekal, Johanna Nel, Sonia Malczyk, Linda Drummond, Nelia P. Steyn

**Affiliations:** 1 Division of Cellular, Nutritional and Physiological Sciences, University of Cape Town, UCT Medical Campus Anzio Road, Anatomy Building, Observatory 7925, Cape Town, South Africa; soniamalczyk@gmail.com (S.M.); linda@linda-drummond.com (L.D.); Nelia.steyn@uct.ac.za (N.P.S.); 2 Department of Logistics, Stellenbosch University, Stellenbosch 7600, South Africa; jhnel@sun.ac.za

**Keywords:** dietary intake, micronutrients, children 1- to <10 -years-old, food fortification, South Africa

## Abstract

In 1999, the National Food Consumption Survey found serious risk of dietary deficiency for a number of micronutrients in 1- to 9-year-old children in South Africa. To address these shortfalls, fortification with vitamin A, thiamine, riboflavin, niacin, vitamin B6, folic acid, iron and zinc of maize meal and bread flour was made mandatory in 2003. The aim of this study was to examine micronutrient intakes of 1- to <10-year-old children after nearly 20 years of fortification in two of the most urbanized and economically active provinces, Gauteng (GTG) and the Western Cape (WC). A multistage stratified cluster random sampling design and methodology was used. Households were visited by fieldworkers who interviewed caregivers and obtained dietary intake data by means of a multiple-pass 24-h recall. Two additional 24-h recalls were completed among a nested sample of 146 participants to adjust the single 24-h recall data of the total sample using the National Cancer Institute Method. Results show that median intake of all the fortification nutrients were above the estimated average requirement (EAR), with the only concern being folate in the WC. Between a quarter and a third of children in the WC, where maize porridge intake was significantly lower than in GTG, had a folate intake below the EAR. Nutrients that are not included in the fortification mix that remain a serious concern are calcium and vitamin D, with intake of dairy and vitamin D sources being very limited in both provinces. The improvement in micronutrient intakes of children is encouraging, however the outstanding nutrient deficiency risks need attention.

## 1. Introduction

Micronutrient malnutrition caused by a deficiency of one or more essential micronutrients is prevalent globally, with children and pregnant and lactating women being the most vulnerable. It appears to be most common in low- and middle-income countries and contributes greatly to the global burden of poverty and disease with low vitamin A, iron and zinc remaining serious risks of deficiencies [1].

In 1999 the National Food Consumption Survey (NFCS) found a serious dietary risk of deficiency for calcium, iron, zinc, folate, vitamin C, vitamin A, vitamin E, riboflavin, niacin, and vitamin B6 in 1- to 9-year-old children in South Africa (SA) [2]. A large percentage of these 1- to 9-year-olds were found to have intakes of less than 67% of the recommended dietary allowance (RDA), which was the criterion for risk of inadequate intake at the time [3]. The diet largely comprised refined carbohydrates and was low in whole grains, dairy products, fruit and vegetables.

In order to combat micronutrient malnutrition and food insecurity in South Africa, the government introduced numerous initiatives, including national food fortification, iodization of salt, and a National School Nutrition Program (NSNP) [4]. The NSNP provides a mid-day school meal comprising a carbohydrate, protein and fruit or vegetable to learners in quintile 1–3 public schools, which are the 60% poorest schools in South Africa [5]. Other initiatives have included iron, folate and vitamin A supplements to pregnant women and children from 6 to 59 months old [4].

In 2003 the Department of Health made large scale food fortification, with vitamin A, thiamine, riboflavin, niacin, vitamin B6, folic acid, iron, and zinc, of maize meal and bread flour, mandatory [6]. This decision was largely based on the outcomes of the NFCS [2]. The NFCS took place nearly 20 years ago and no follow-up of children of the same age has been undertaken. Thus, changes in diet and micronutrient intake of children over this period are largely unknown. However, some local studies have indicated that certain micronutrient intakes may still be low [7]. Since vitamin C, vitamin D, vitamin E and calcium are not added to the fortification premix, it is also not known if the intake of these nutrients has improved.

In 2018 a provincially representative dietary intake study (PDIS) of 1- to <10-year-old children was undertaken in Gauteng (GTG) and in the Western Cape (WC) [8]. These are regarded as being the two most economically active and urbanized provinces in South Africa. Results from this study indicated that 20.1% of children in the two provinces experienced hunger [8]. This was considerably lower than that found in 1- to 9-year-olds in 1999 (41.8 in GTG, 31.3 % in WC) [9] and implies that food security and malnutrition have improved in South Africa. Furthermore, nutritional status by means of anthropometry was determined in the PDIS [8]. In children under five years old 21.6% were found to be stunted, 10.3% overweight and 7.0% obese. In 5- to <10-year-olds 6.7% were stunted, 13.4% overweight and 6.8% obese. Stunting and overweight in the same child was present in 5.7% under-five year olds and 1.7% in 5- to <10-year-olds. These results reflect the double burden of malnutrition still found in children. The aim of this study was to present findings on micronutrient intakes of children, main food sources of micronutrients and per capita daily intake of specified food groups.

## 2. Materials and Methods 

### 2.1. Study Area

The two provinces selected were Gauteng (GTG) and the Western Cape (WC), because they are the most rapidly urbanizing and wealthiest provinces with extensive migration from rural areas to cities in search of jobs and a better quality of life [10].

### 2.2. Structure of the Sample and the Sampling Procedure 

Six strata were identified during the design phase, namely two provinces (GTG and WC), with each having three areas of residence: urban formal, urban informal and rural areas. Formal areas include planned developments with roads, infrastructure and brick houses. Informal areas are unplanned developments with housing not made from formal building materials, also referred to as shacks. A rural area is any area that is not classified as urban, and may comprise a tribal area, commercial farm or an informal settlement, and is so designated by Statistics South Africa [11]. All the enumerator areas (EAs) were identified in each stratum. A stratified two-stage sample design was used with a probability proportional to size sampling of EAs at the first stage, and systematic sampling of households within the EAs at the second stage.

The number of households per sampling stratum (province and residential area), taking non-response into account was calculated to be N (= 175); where the design effect (Deft = 1.3), the estimated proportion of children classified as stunted (*p* = 0.21), and the desired relative standard error (a = 0.2) are based on estimations from previous surveys. The *p* value for stunting was used since the current study formed part of a larger study which also looked at anthropometric status [8]. The individual response rate (R_1_ = 0.96) was expected to be higher than the expected household gross response rate (R_2_ = 0.89). The number of eligible individuals per households (d = 1.06) was calculated as the average number of children aged 1 to <10-years per household. It was proposed to survey 175 × 6 strata, or 1050 households. 

For the precision of estimates to be acceptable across regions, experience shows that a minimum of 50 interviews per stratum are needed so that reliable estimations for indicators under investigation can be obtained. The final sample allocation reflects a power allocation of 0.5, which is between the proportional allocation and the equal size allocation, so that the survey precision in the urban formal areas is comparable with the urban informal and rural areas, with urban informal and rural areas slightly over-sampled. Since the sample sizes of GTG rural, WC rural and urban informal were less than 150, we increased sampling accordingly to ensure sufficient observations per cell in each age group, with the proposed sample size then being 1050 + 218 = 1268. A total of 84 EAs were selected from the six strata, 25 formal residential, 10 informal residential and 11 rural EAs in GTG, and 18 formal residential, 10 informal residential and 10 rural EAs in the WC.

### 2.3. Selection of Households 

Maps of relevant primary sampling units were generated and passed on to the respective fieldwork teams. An estimate was made of the total number of households (HHs) in each EA to determine the approximate number of qualifying HHs with children within the prescribed age interval in the EA. A listing of eligible households was compiled in all selected EAs, which served as a sampling frame for the selection of households. HHs (a maximum of 16) were then selected based on a predetermined fixed interval (calculated to be specific to each EA) starting from a randomly determined point. A backup sampling frame was constructed in each EA by asking members of the 16 selected HHs to identify nearby HHs with women and children of the appropriate age.

### 2.4. Selection of Children within Households

One child in each randomly selected HH was included in the survey. If there was more than one child present in the prescribed age interval within a HH, then all eligible children in the HH in age order were numbered for random selection of one child using a “Random Number Table” designed for this purpose. 

The inclusion criteria for the current study were as follows: children aged 1 to <10 years (12–119 months) old; male or female; availability of a parent/primary caregiver to provide consent; and availability of a parent/primary caregiver to assist with completion of the research questionnaires. The exclusion criteria were as follows: children who were mentally or physically handicapped; children who were on a prescribed diet for a childhood disease e.g., Type 1 diabetes, phenylketonuria and other conditions; children who were ill at the time of the visit or were ill during the past 24 h; children whose mothers/caregivers were unable to respond, or appeared to be incapable of responding or providing reliable information; children whose mother/caregiver was under the influence of alcohol/drugs or was under 15 years old.

Sampling weights were calculated to adjust for the oversampling in the rural and urban informal areas and the number of children in the 1 to <3, 3 to <6, and 6 to <10-years age groups, bearing in mind the survey design. The final weight was the product of the proportional and realization weights. The final post-hoc stratification weighting reflects the census population of the Western Cape and Gauteng provinces. The three age groups were demarcated to reflect children who are in the first 1000 days of life (children in their third year, but not yet three years old, were included in this group), older preschool children and primary school aged children. 

### 2.5. Fieldwork Teams

Fieldwork in each province was led by a registered dietitian (fieldwork coordinator) who was responsible for the overall management of the research teams in the two provinces. Both GTG and the WC had two research teams each including a team leader and two pairs of field workers for a total of 11 team members per province. The field workers were selected based on a minimum level of grade 12 i.e., completion of high school, as well as other experience in surveys and in field work. 

Before data collection began, team leaders and field workers received a week-long extensive training session, according to a manual which had been developed for the purpose of the study. Training was facilitated by researchers experienced in administration of sociodemographic and dietary questionnaires, as well as the fieldwork coordinators (registered dietitians). Training of the WC fieldwork teams took place in Cape Town and was attended by the GTG fieldwork coordinator. Subsequently, training of GTG fieldworkers took place in Johannesburg which was co-facilitated by the WC fieldwork coordinator to contribute to data fidelity. After each training module the field workers practiced using the questionnaires through role play sessions with each other. At the end of the week the field workers took a practical and written test based on case studies. Field workers who did not achieve a certain percentage were not selected. Field workers carried their manuals in the field during the period of the study. 

### 2.6. Measures

#### 2.6.1. Socio-Demographic Questionnaire

The questionnaire comprised questions about the child: birth date, gender, birth order, schooling/day care centre, and dietary supplements from clinics. Questions about the family and household: head of household, primary caregiver, marital status of mother, education and employment status of mother and father, type of house, availability of electricity or other energy devices, drinking water, type of toilet, and household density. These variables were selected as they were used in the NFCS and many were found to be significant predictors of nutritional status [12].

A wealth index was calculated as indicated by the World Bank [13] and applied in the 2016 South Africa Demographic and Health Survey [14]. Principal component analysis was used to estimate relative wealth, and this estimation is based on the first principal component. This component contributes to a wealth index that assigns a larger weight to assets that vary the most across households, so that an asset found in all households is given a weight of zero. The wealth index was based on amenities available in the home and environment and was developed using an iterated principal factor analysis (Supplementary Table S1).

#### 2.6.2. Hunger Scale Questionnaire

Hunger (food security) was measured using the Community Childhood Hunger Identification Project (CCHIP) questionnaire [15], included as Supplementary Table S2. This questionnaire measures household, child and individual level food security. Altogether, there are eight questions in the scale. If any of these are affirmative, then a score of one is given. A total score of 5–8 indicates that a food shortage is present in the house. A score of 1–4 indicates that the household is at risk of hunger (poor food security) and a score of zero indicates that the house is food secure. These scores were used to calculate an association with selected dietary variables.

#### 2.6.3. Dietary Intake

A 24-h recall was undertaken with each participant to determine micronutrient intakes. Literature indicates that accuracy of reporting own dietary intake in younger children is not good but improves between the ages of 8 and 12 years [16]. Consequently, in this study all dietary interviews took place in the presence and with the input of the mother/primary caregiver. For 1- to 6-year-old children the mother/caregiver reported on the intake of the child on the previous day with no input from the child. For 6- to <10-year-old children the mother/caregiver and child were interviewed together to record the dietary intake during the prior 24 h. If the child had been at a day care centre the previous day, the centre was visited by the fieldworker and the meals and portion sizes determined for the 24 h in question. All weekdays and Sundays were covered proportionally by each team to ensure that potential variation because of day of the week was captured.

A single 24-h recall has been used as the primary instrument for measuring dietary intake in numerous large dietary studies. A common concern with a single 24-h recall is the day-to-day variation in the diet of free-living populations. The magnitude of the mostly random within-person variance varies by nutrient and is largely dependent on cultural and ecological factors. Methodological challenges in estimation of dietary intake may also contribute to within-person error. These errors result in large standard deviations (SDs) in population groups and insignificant regression coefficients. Another important result of exaggerated variation is that the percentage of subjects below or above specified cut-points will be distorted. The National Cancer Institute (NCI) method [17,18] that was developed to distinguish within-person from between-person variation, accounts for extreme intakes, including zero intake, and allows for adjustment for covariates and association analyses, was applied in this study to estimate the usual dietary intake from repeated 24-h dietary recall assessments on a subsample of 148 (2nd recall) and 146 (3rd recall) children.

For logistical reasons, this subsample was recruited from the last five EAs visited in each province. The same houses were revisited, and the same children and caregivers interviewed. Comparison of sociodemographic variables between those who completed one 24-h recall and those who completed repeated recalls showed only two significant differences. The subgroup had more unmarried mothers than the total group (*p* < 0.001) and more black African children (*p* < 0.001) (Supplementary Table S4). Whether the 24-h recall was less, the same or more than the child’s usual intake was also recorded for the total group and the two additional recalls completed for the subgroup and considered in the application of the NCI method.

We further examined the transformation parameters from the BOXCOX_SURVEY macro when transforming the variables to approximate normality, to determine whether the results for each nutrient was sensitive to the transformation applied. A larger mean bias is reflected by a lambda value obtained from the Box–Cox transformation of less than 0.15 [19]. The contribution this type of correction makes to estimation of usual dietary intake from a single 24-h recall is illustrated by the work of Piernas et al. 2015 [20] who applied the NCI method to correct the first 24-h dietary recall collected in a sample of 6- to 47.9-month-old children (*n* = 2045) with a second 24-h recall collected in a sub-sample (*n* = 178). They found a reduced variability for all estimates across all age groups for usual intake data for energy, fat, iron and zinc. Moreover, results from the uncorrected single recall tended to overestimate inadequate iron and zinc intakes in 1 to <3-year olds (%<estimated average requirement (EAR) for iron 18% versus 1% and % < EAR for zinc 13% versus 1%) and 3- to <6-year olds (% < EAR for iron 7% versus 1% and % < EAR for zinc 6% versus 1%).

The multiple pass method of the 24-h recall was used to administer the 24-h recall [21]. Essentially the interviewer first went through the previous day’s intake by recording all the food items and drinks that were consumed between waking up in the morning until going to sleep in the evening (and during the night if applicable). Recall was helped by the interviewer going through the daily activities with the participant and linking them to eating occasions. Next, the interviewer prompted the respondent to identify food and drinks that may have been “forgotten,” such as cold drinks, candies and snacks. Information was then recorded regarding when and where the various food items were consumed. Following this, more detailed information was obtained regarding the preparation of the foods and individual ingredients as relevant. Lastly, portion sizes were recorded as accurately as possible for all foods/drinks consumed. A combination of methods was used to determine mixed dishes. When generic recipes were available in the food composition tables, they were used. When no recipes were available, the ingredients were calculated proportionally and added as individual items from the food composition tables. Field workers were taught how to do this during their training programme.

**Figure 1:**
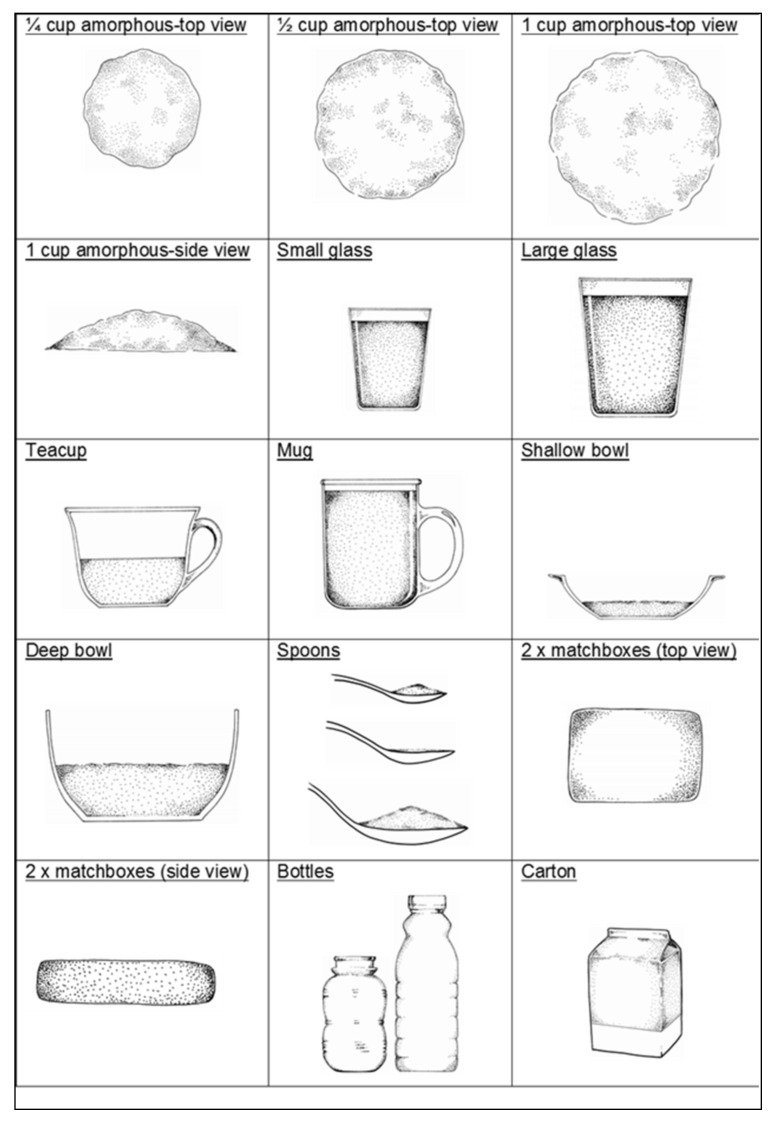
Examples of life-size sketches and measures used in the study.

Portion sizes were obtained using a booklet adapted from the Dietary Assessment and Education Kit (DAEK) [22]. The booklet comprises life-size sketches of generic household utensils and crockery (Figure 1) and life size portions of actual foods e.g., different slices of bread varying in size and thickness, to make estimations of portion size as accurately as possible. The sketches have been validated in adolescents [23]. Generic three-dimensional food models made from flour were also used to assist in recording volume measures such as porridge and rice.

Breast milk consumption was quantified by asking mothers whether their child was still receiving breast milk and if yes, the number of feeds the child received during the previous 24 h. Based on the study by Neville et al. [24] we used an estimate of 100 mL per feed to calculate the volume of breast milk consumed per day.

#### 2.6.4. Anthropometry of Mothers

Detail of the weight and height measures of mothers is described elsewhere [8,25]. Body mass index (BMI) was calculated for the mothers as weight divided by height squared (kg/m^2^) and classified as underweight (<18.5), normal weight (18.5–24.9), overweight (25–29.9) or obese (≥30) [26].

### 2.7. Data Analyses

After completion of an EA, the questionnaires were checked by the two provincial dietitians who managed the fieldwork and the quality control of data collection in each province. The questionnaires were then dispatched to a central point for data entry. Data analyses were conducted using SAS Version 9.4, SAS for Windows (SAS Institute, Carry, NC, USA). Weighted means, proportions and 95% confidence intervals were calculated by incorporating the complex survey design. Frequencies were tallied for all the socio-demographic variables which were compared between the WC and GTG using the Rao–Scott chi-square test.

The 24-h recalls were coded by the two fieldwork coordinators (registered dietitians). Where necessary codes were confirmed in consultation with the rest of the research team to ensure uniform decisions and coding of foods. Dietary data were analysed using the South African Food Composition Tables (SAFCT) [27]. Mean (95% confidence intervals) and median (inter quartile range) intake of energy and micronutrients were calculated as well as the prevalence of children with intakes less than the EARs) [28]. The EAR cut-offs for different micronutrients are included in Table 2, Table 3 and Table 4. Of note is that for 16% of food items included in the SAFCTs the vitamin D content values are not yet known (missing) resulting in unavoidable underestimations of dietary vitamin D intake (personal communication with Ms. J Chetty of the SAFCT division, South African Medical Research Council (SAMRC). Furthermore, we did not attempt to estimate sunshine exposure of the children. Vitamin D synthesis occurs on exposure to ultraviolet B rays which can contribute significant quantities of vitamin D [29]. Results on the adequacy of vitamin D intake should therefore be interpreted with caution.

We identified the percent of individual food items which contributed at least 5% to selected micronutrients for the total sample of children. This was done in the NFCS [2,9] and helped to provide important information on foods selected to be fortified at the time. Additionally, we calculated the mean (95% confidence intervals) daily per capita intake in grams from 16 food groups derived from the food group classification introduced by the Food and Agriculture Organization (FAO) and Food and Nutrition Technical Assistance Project (FANTA) [30]. Our adaptation of the FAO–FANTA food groups focused on providing insights in food groups that contribute to the intake of particular minerals and vitamins. These groups were 1) bread, 2) maize porridge (both fortified by law in South Africa), 3) breakfast cereals fortified commercially, 4) other starchy foods (including potatoes and sweet potatoes), 5) legumes and nuts, 6) dairy, 7) cheese, 8) fats and oils (including olives and avocado) 9) eggs, 10) liver, 11) canned pilchards/sardines, 12) flesh foods (red meat and poultry), 13) vitamin A (b-carotene) rich fruit and vegetables, 14) other fruit, 15) other vegetables and 16) all remaining food items (breast milk substitutes, tea/coffee, commercial soups, sauces, sugar sweetened beverages, sweets and chocolates, jam and syrup, sugar and condiments). Exclusion of potatoes, sweet potatoes, olives and avocadoes from the vegetable group and allocation to the starch and fats and oils groups is in line with recommendations by Naude [31].

The independent t-test was used to test for significant differences of mean nutrient intakes between the two provinces. The standard errors used for the independent t-tests required additional programming to implement the balanced repeated replication (BRR) when using the NCI method (see Supplementary Table S3 for more detail) [17,18].

Socio-demographic predictors of having eaten maize porridge (yes) and bread (white or brown) (yes) were identified by firstly conducted bivariate logistic regression within each age group, considering the complex sample design, with the following independent variables: who looks after the child; age and gender of the child; head of the household; marital status of the mother; mother’s employment status; father’s employment status; mother’s education level; father’s education level; BMI of the mother of the child; wealth index quintile; province; type of residence; ethnicity and risk of hunger. Significant relationships (*p* < 0.05) found with the bivariate regression analyses were further investigated using a multivariate logistic model. Odds ratio estimates with 95% confidence intervals are reported for the and multivariate logistic regressions only. The Wald Chi-square was used to test the significance of the estimates in the logistic regression. Multicollinearity was addressed by excluding highly correlated variables.

Since no significant differences were found for mean energy and micronutrient intakes between areas of residence or gender in both provinces, the data was pooled and is presented by age group and province.

Direct comparison between the 1999 NFCS intake results for 1- to 9-year-olds and the 2018 PDIS data for 1- to <10-year-olds was complicated by the difference in age group demarcation between the two studies. Furthermore the 1999 NFCS data set was not adjusted using repeated 24-h recalls in a sub-sample as was done in the 2018 PDIS. To provide some broad insights in change in intakes of micronutrients over time we extracted the median intakes for each age group in GTG and the WC from the NFCS report [9]. Median intakes for the NFCS age groups (NFCS age groups: 1–3 years, 4–6 years and 7–9 years; PDIS age groups: 1 to <3 years, 3 to <6 years and 6 to <10 years) were generated from the 2018 PDIS data set and depicted with the NFCS results and EAR for each nutrient.

### 2.8. Ethics

The study was conducted in accordance with the principles of the 2013 Declaration of Helsinki [32], Good Clinical Practice (GCP) and the laws of South Africa. The approval from the Faculty of Health Sciences Human Research Ethics Committee at UCT was obtained on 18 July (HREC REF: 326/2018). Parents or primary caregivers of children provided informed, signed consent. Children aged 6- to <10-years-old were also asked for verbal assent.

## 3. Results

Table 1 presents general sociodemographic and other characteristics of the sample. The mother looked after the child most of the time as the primary caregiver (70%) followed by a grandparent being the most likely alternative primary caregiver (18%). The latter was significantly more likely in the WC than in GTG. The father was the head of the household in 40% of homes, followed by the grandmother (24%). Overall 53% of mothers had not completed grade 12, with mothers in WC being significantly more likely to have a grade 12 level of education. Fewer mothers were employed (28%) compared with fathers (65%). Overall 75% of the sample were black African and 24 % were of mixed ancestry and 88% of the sample lived in urban formal areas. Twenty-five percent of mothers were overweight and 43% were obese, with mothers in the WC being significantly more likely to be obese. Twenty-four percent of households were at risk of hunger and 21% had a food shortage in the home.

**Figure 2:**
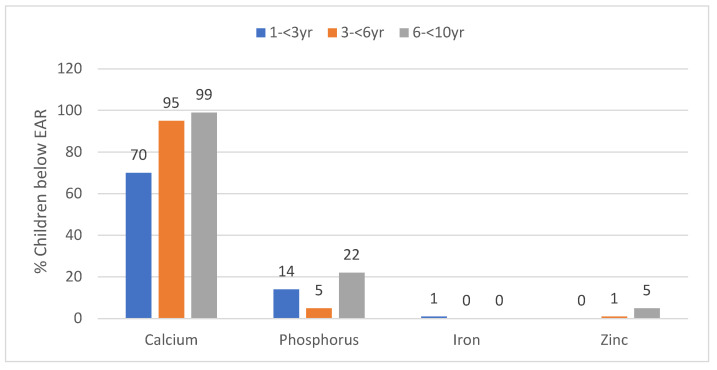
Percent children in the three age groups with values below the estimated average requirement (EAR) for minerals (taken to nearest decimal)**.**

In Table 2 the mineral intake values are presented by province and age group. Median calcium intake was less than the EAR in all three age groups, with the prevalence of intake below the EAR increasing from 70.2% to 99.4% with age (Figure 2). Median phosphorus intake was above the EAR in all three age groups and less than 20% of the children had a phosphorus intake below the EAR (Table 2, Figure 2). Median iron intake was above the EAR in all three age groups and only 1% or less of the children had an intake below the EAR (Table 2, Figure 2). Median zinc intake was above the EAR in all three age groups and less than 1% in the two youngest age groups and 4.9% in the older age group had and intake below the EAR (Table 2, Figure 2). The daily intake of calcium was significantly lower in GTG than in the WC in the 3- to <6-year-old and the 6- to <10-year-old groups. Iron and zinc intakes were significantly higher in GTG than WC for the 6- to <10-year-olds.

**Figure 3:**
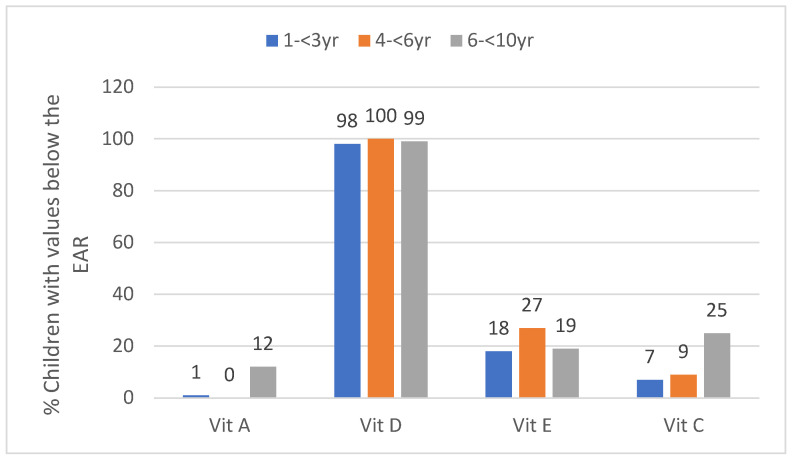
Percent children in the three age groups with values below the estimated average requirement (EAR) for vitamins A, D, E and C (taken to nearest decimal).

Median vitamin A intake was above the EAR in all three age groups and the percentage with an intake below the EAR was the highest in the oldest age group (12%) (Table 3, Figure 3). Median vitamin D intake was well below the EAR in all three age groups, with almost a 100% in the youngest and oldest age groups and 100% in the middle age group having an intake below the EAR (Table 3, Figure 3). Median vitamin E intake was above the EAR in all three age groups, with just over a quarter of the 3 to <6-year-olds having an intake below the EAR and almost 20% of the youngest and oldest groups having an intake below the EAR (Table 3, Figure 3). Median vitamin C intake was above the EAR in the two younger age groups, but below the EAR in the 6- to <10-year-olds, with a quarter of the latter group having an intake below the EAR (Table 3, Figure 3). Vitamin C intake was significantly lower in GTG than the WC in all age groups (Table 3). 

**Figure 4:**
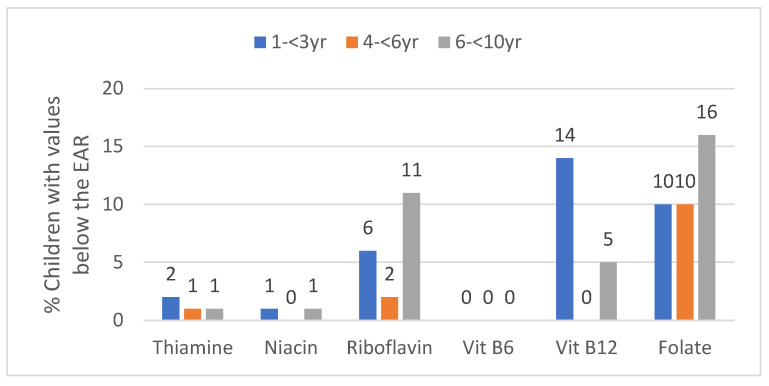
Percent children in the three age groups with values below the estimated average requirement (EAR) for B vitamins (taken to nearest decimal).

Median thiamine, niacin and vitamin B6 intake was above the EAR in all three age groups and less than 2% in each age group had an intake below the EAR for these nutrients (Table 4, Figure 4). Median riboflavin intake was also above the EAR in all three age groups with the percentage below the EAR being the highest in the oldest age group (11.4%) and the lowest in the middle age group (2.3%) (Table 4, Figure 4). Median vitamin B12 intake was above the EAR in all three age groups. None of the 3- to <6-year-olds had an intake below the EAR, while 14.3% of the youngest age group and 5.4% of the oldest age group had an intake below the EAR (Table 4, Figure 4).

Median intake of folate was above the EAR in all three age groups. One in 10 of the 1- to <3-year-olds, as well as 3- to <6-year-olds had an intake below the EAR and 15.5% of the 6- to <10-year-olds had an intake below the EAR. Thiamine and folate intakes were significantly higher in GTG than WC in all age groups. In the WC almost a quarter of the two younger age groups and a third of the 6- to <10-year-olds had an intake of folate below the EAR.

Riboflavin intake was significantly lower in GTG than the WC in the two older groups, while the intake of vitamin B6 was significantly higher in GTG than in the in the two older groups. Vitamin B12 intake was significantly lower in GTG than WC in the two younger age groups, with almost a fifth of the 1- to <3-year-olds having an intake below the EAR (Table 4, Figure 4).

Table 5 provides results on food items contributing at least 5% of total intake of calcium, iron and zinc in the total sample. Whole milk provided about a quarter of the calcium, followed by maize porridge, canned pilchards/sardine, maas (a traditional sour drink made from maize) /sour milk, breast milk substitute (BMS) and yoghurt. Maize porridge contributed a quarter of the iron, followed by white bread, brown bread and high fiber cereal. Maize porridge contributed more than a quarter of the zinc followed by brown bread, white bread, beef and chicken. 

Table 6 provides data on food items contributing at least 5% of total intake of vitamins A, D, E, C and the B vitamins. Organ meat and maize porridge each contributed approximately a quarter of the vitamin A, followed by medium fat margarine and whole milk. Canned pilchards/sardines and eggs each contributed approximately a quarter of the vitamin D followed by vitamin A-rich vegetables and medium fat margarine. Polyunsaturated fatty acids (PUFA) oil contributed almost a fifth of the vitamin E, followed by medium fat margarine, maize porridge, salty snacks, vitamin C vegetable group and eggs. Fruit juice contributed about a fifth of the vitamin C followed by potato/sweet potato, vitamin C group vegetables and maize porridge. 

Organ meat and canned pilchards/sardines each contributed almost a third of the vitamin B12 followed by whole milk and beef. Contributors to riboflavin intake were maize porridge (less than a fifth), followed by whole milk, high- and low-fiber cereals and organ meats. Maize porridge contributed almost half of the folate followed by brown bread, white bread, and organ meats. Maize porridge contributed more than a third of the thiamine followed by white bread, brown bread and high-fiber cereal. Maize porridge and chicken each contributed almost a fifth of the niacin, followed by white bread, brown bread, high fiber cereal and potato/sweet potato. White bread and maize porridge each contributed approximately a quarter of the vitamin B6, followed by brown bread (a fifth) and potato/sweet potato.

The mean (CI) per capita intake of children in the three age groups of the 16 food groups adapted from the FAO–FANTA groups [30] is presented in Table 7. Next to the ‘All other’ food group, maize porridge contributed most to total food intake in grams in all three age groups, followed closely by intake of other starchy foods (excluding bread and fortified cereals). Maize porridge intake was significantly higher in GTG than the WC in all three age groups, while the intake of other starchy foods was significantly lower in GTG than in the WC in all three age groups. Bread intake was lowest in the youngest age group and did not differ between the two provinces. 

Dairy intake reduced with age and was significantly lower in GTG than the WC in all three age groups. The intake of flesh foods was the lowest in the youngest age group and significantly lower in GTG than the WC in all three age groups. Egg intake ranged from 8–10 g across the age groups and did not differ between the two provinces. Canned pilchard/sardine intake ranged from 6–12 g across the three age groups, with intake being significantly higher in GTG than the WC in the youngest age group only. Intake of cheese and liver was less than 5 g in all three age groups and did not differ between provinces. 

Total fruit and vegetable intake (Fruit and Vegetables: Vitamin A-rich group + Other vegetables group + Other fruit group) was approximately 110 g in all three age groups and the intake of ‘other’ vegetables was significantly higher in GTG than the WC in the two older age groups.

In Table 8 predictors for having eaten bread and maize porridge are shown (only results of the multivariate logistic regression models). Significant predictors of having consumed bread in the 24-h recall period include older age, being in an ethnic group other than black African or mixed ancestry and living in rural areas. For having consumed maize porridge being in the highest wealth index quintile and being of mixed ancestry predicted not having consumed maize porridge, while living in GTG and having a food shortage in the house predicted having consumed maize porridge.

Median intakes for the investigated micronutrients derived from the 1999 NFCS report [32] and 2018 PDIS data according to the NFCS age groups compared to the EAR for each nutrient are depicted in Supplementary Figures S1–S3. Median calcium and vitamin D intakes were below the EAR in all age groups in 1999 and seem to not have improved over the years. Median iron and zinc intakes were above the EAR at both time points in all age groups, with substantial increases in intakes evident in both provinces in all age groups for both these nutrients from 1999 to 2018. Median folate intake was above the EAR in the two older age groups in the WC, but below it in GTG in 1999. 

A substantial improvement in folate intake from 1999 to 2018 is evident for children in GTG in all three age groups, while this is not the case in the WC. Median vitamin E intake was below the EAR in all age groups in both GTG and the WC in 1999, with all medians improving to above the EAR in 2018. Vitamin C intake was below the EAR in Gauteng and above it in the WC in 1999 in all age groups, with all median intakes being above the EAR in 2018. Median intakes of thiamine, riboflavin, niacin, vitamin B6 and vitamin B12 were above the EAR at both time points in all age groups, with the exception of niacin being below the EAR in the 1–3-year-olds in GTG in 1999. Thiamine, niacin and vitamin B6 intakes seem to have increased in both provinces in all age groups. Riboflavin and vitamin B12 intakes seem to have increased in children in GTG, but not in the WC. 

## 4. Discussion

Results of the 2018 PDIS in GTG and the WC show that median intakes were above the EAR and risk of deficiency is negligible for most micronutrients in 1- to <10-year-old children, with the exceptions being calcium and vitamin D in all three age groups in both provinces, and to some extent folate, vitamin C and vitamin E in select age groups and provinces. 

Fortification of maize meal and bread flour with vitamin A, thiamine, riboflavin, niacin, vitamin B6, folic acid, iron and zinc became mandatory in 2003 [6] largely as a result of the outcomes of the 1999 NFCS that showed that 1- to 9-year-old children in South Africa were at risk of deficiency of these six micronutrients [2]. Other studies in African children have shown similar dietary risks e.g., inadequate intakes and biochemical levels reflecting deficiency in 4- to 8-year-old rural Zambian children for iron, folate, vitamin B12 and calcium that was proposed to be the result of a monotonous, predominantly plant-based diet [33]. For 5- to 17-year-olds in urban areas of Senegal, inadequate intakes of dietary iron, folate, vitamin B12 and calcium were reported, especially in the older children and attributed to a diet poor in dairy products, meat, fruit and vegetables [34]. No interventions were put in place at the time to address the risk of deficiency of calcium and vitamins C, D and E that was also evident at the time [2]. 

The contribution of maize porridge and bread (brown or white) to fortification nutrient intakes in the 2018 PDIS is evident from the fact that these foods were the top two or three sources of iron, zinc, folate, thiamine, niacin and vitamin B6, as well as one of the top three sources of vitamin A and riboflavin. Swanepoel et al. [35] also reported that children at ages 12 and 18 months consumed fortified maize meal and bread (although to a lesser extent) that contributed to micronutrient intake. At 18 months, fortified staples contributed >30% of iron, zinc, vitamin A, thiamine, niacin, vitamin B6, and folate in this cohort of children. 

Consideration of the per capita intake of maize porridge (203 g to 198 g from the youngest to the oldest age group respectively) and bread intake (16 g to 67 g from the youngest to the oldest age groups respectively) illustrate that maize porridge was most probably the most important fortification vehicle when compared to bread. The per capita maize porridge intake across the groups and provinces in the 1999 NFCS (341 g) [36] was also higher than bread intake (69 g) and thus similar to intakes recorded in the 2018 PDIS. Maize porridge intake of 7- to 11-year-old learners in a peri-urban informal settlement in GTG was found to be 628 g and bread intake 85 g (based on a quantified food frequency) [37]. The lower intake of especially maize porridge in the 2018 PDIS may be linked to the fact that where the 1999 NFCS covered all nine provinces in the country, including less urbanized provinces, GTG and WC are the most urbanized and thus advanced in terms of the nutrition transition. The differences between 1–6-year-olds living in more rural areas versus more urban provinces in the country in the intake of maize porridge and bread is also evident from the work by Faber et al. [38] in four provinces in South Africa. These researchers reported that 81% of the children in rural KwaZulu-Natal ate maize porridge and 64% bread, 98% of the children in rural Limpopo consumed maize porridge and 49% bread, 60% of children in urban Northern Cape ate maize porridge and 62% bread and 31% of the children in urban WC ate maize porridge and 71% bread. 

Of note is that the per capita intake of maize porridge in the 2018 PDIS was significantly lower in the WC than in GTG in all three age groups. Our regression analysis shows that being in the highest wealth index quintile and being of mixed ancestry predicted being less likely to have consumed maize porridge, while living in GTG and having a food shortage in the house predicted being more likely to have consumed maize porridge. This may be due to both socioeconomic and cultural differences. The majority of the randomly selected children in GTG were black African (98%) and maize porridge forms part of their traditional diet while also being a more economical staple than bread [39]. The majority of randomly selected children in the WC were from mixed ancestry (68%) and maize porridge is not traditionally consumed as starchy staple by these South Africans [39]. 

Unlike maize porridge, bread intake did not differ between the two provinces for any of the three age groups. Significant predictors of having consumed bread include older age, which is also evident from the increase in per capita intake of bread from approximately half a slice of bread in the 1- to <3-year-olds, just more than a slice in the 3- to <6-year-olds and two slices in the oldest age group. Although being in an ethnic group other than black African or mixed ancestry emerged as a predictor of bread intake, the ‘other’ group comprised only 4% of the total sample and these results should thus be interpreted with caution. 

It is interesting to note that living in rural areas was a predictor of having eaten bread. As is indicated in the methods section, a rural area was defined as an area not classified as urban, including tribal areas, commercial farms or informal settlements [11]. It could be argued that bread intake, especially fortified bread, would be limited in tribal areas where intake of starchy staples such as maize may be more common [38]. Access to fortified bread flour and bread as such seems more likely on commercial farms and in informal settlements close to urban areas. This is in line with reports that one element of the nutrition transition involves increased consumption of white bread [39]. Secondary analysis of data on the foods most commonly consumed by South African adults [39,40] showed that per capita white bread intake was 35g and brown bread intake 101 g for rural dwellers, while this was 73 g and 58 g, respectively, for urban dwellers.

With the significantly lower intake of fortified foods by children in all three age groups in the WC than in GTG, one would expect significantly lower intakes of fortification nutrients and greater risk of deficiency in the WC. Some differences were evident, but the only material difference involved folate, where the intake of children in the WC was significantly lower than in GTG in all three age groups. The percentage below the EAR was also substantially higher in the WC than GTG i.e., 1- to 3-year-olds: 23% versus 3%; 3- to <6-year-olds: 23% versus 4%; and 6- to <10-year-olds: 32% versus 7%. Folate is a donor of 1-carbon moieties and is essential for nucleotide biosynthesis [41]. Key sources other than fortified maize porridge and bread are liver (chicken liver: 770 mcg/100 g, sheep liver: 400 mcg/100 g and beef liver: 220 mcg/100 g), dark green leafy vegetables (146 mcg/100 g); legumes (between 100–180 mcg folate/100 g depending on the type) and fortified breakfast cereals (250 mcg/100 g) [27,41]. 

Results on the per capita intake of these foods within each age group did not differ between provinces and show that they most probably made a negligible contribution to total folate intake. Legume intake ranged from 9 g to 17 g which is similar to the per capita intake reported for the total sample in the 1999 NFCS of 13 g [36]. Per capita intake of fortified cereals was less than 10 g and liver less than 2 g in all three age groups in the 2018 PDIS (there is no comparative value for the NFCS for these two foods). Green leafy vegetables were combined with other vitamin A-rich vegetables e.g., carrots, pumpkin and butternut in our FAO–FANTA groupings [30], but the total per capita intake of this combination of vegetables was less than 25 g in all three age groups in both provinces with the oldest group having the lowest intakes. It could thus be argued that as far as folate is concerned, children who do not typically eat a combination of 250–350 g maize porridge and bread per day may be at increased risk of folate deficiency if they do not consume other key sources on a daily basis. 

Results show that iron and zinc intakes were similar between the two provinces in the two younger age groups, but lower in 6- to <10-year-olds in the WC than in GTG. However, percentage intake below the EAR for iron in all three age group was less than 2% in both provinces, indicating that there seems to be no reason for concern about iron intakes. Improvements in iron status in children in South Africa is also reflected in the decrease in prevalence of iron deficiency anemia (Hb <11.5 g/dL) from 28.9% in 2005 to 10.7% in the 2012 [42]. Risk of zinc deficiency also seems to be low with the percentage zinc intake below the EAR being less than 1% in the two younger age groups and less than 10% in the 6- to <10-year-olds. 

Thiamine intake was significantly lower in the WC than in GTG in all three age groups, but the median intake for all three groups was above the EAR and the percentage below the EAR was less than 4% across the age groups. Vitamin B6 intake was also lower in the WC in the two older age groups, but the median intakes were above the EAR and not one child in the study had an intake below the EAR. Interestingly, Mitsopoulou et al. [43] also found that iron, zinc, thiamine, riboflavin, niacin and vitamin B6 intakes were sufficient across similar age groups in the Hellenic National Nutrition and Health Survey (HNNHS), with processed cereals being the main source of all six of these micronutrients in Greek children [43]. 

Although fortified foods were the most important sources of iron, zinc, thiamine, riboflavin, niacin and vitamin B6 in the 2018 PDIS, alternative sources seemed to have made a material contribution to intakes by children in the WC. Good sources of these micronutrients include flesh foods and dairy [27], both of which per capita intakes in the WC were significantly higher (almost double) than in GTG. Mean per capita dairy intake found in the 1999 NFCS was 104 g for the total sample [36], which is was lower than the 251 g, 183 g and 139 g recorded in the 2018 PDIS for the two provinces combined within each age group (1- to <3-year-olds to 6- to <10-year-olds). The intake of eggs, which is also a good source of many of these nutrients, was significantly higher in the 1- to <3-year-olds in the WC than in GTG and tended to be higher in the 6- to <10-year-olds. Mean per capita egg intake in the 1999 NFCS was 8g for the total sample [36], thus very similar to the approximately 10 g recorded in the 2018 PDIS for the two provinces combined within each age group. The per capita daily liver intake, an excellent source of the fortification micronutrients, was below 2 g in all three age groups and did not differ between the provinces. Liver intake in the 1999 NFCS was less than 1 g [36]. Based on this scenario it could be argued that it would be possible to obtain the necessary iron, zinc, thiamine, riboflavin, niacin and vitamin B6 from the diet without eating the amount of maize porridge and bread eaten by children in GTG if children’s diets include sufficient flesh foods, dairy and eggs. 

Flesh foods, dairy and canned pilchards/sardines were also identified as the most important contributors to vitamin B12 intake in the 2018 PDIS. With the lower per capita intake of most of these foods in GTG, it comes as no surprise that vitamin B12 intakes in the two younger age groups were significantly lower in this province than in the WC. However, the intakes in all groups were above the EAR, with only the 1- to <3-year-olds in GTG showing some risk of deficiency in terms of percentage intake below the EAR (GTG: 19 %). 

Vitamin A deficiency in children has long been a concern in South Africa [44], with targeted interventions to address this malnutrition problem including fortification of maize and bread flour with vitamin A and roll out of the national routine vitamin A supplementation of children younger than 6 years at all public health facilities (Vitamin A Supplementation programme) from 2002 onwards [44]. Vitamin A is essential for many functions including normal vision and eye health, immunity, cellular proliferation, differentiation and apoptosis [45]. It is thus encouraging to observe that dietary risk of deficiency seems to have decreased substantially in the 2018 PDIS when compared to the 1999 NFCS [2]. Moreover, there were no significant differences between the two provinces for vitamin A intake in any one of the three age groups, with the median intake being above the EAR and the percentage below the EAR being less than 2% for the two younger age groups and 12% for the oldest age group. Mitsopoulou et al. [43] also found that vitamin A intake below the EAR was more prominent in older children in Greece. 

The improvement in vitamin A status of South African children is also evident from the South African National Health and Nutrition Examination Survey (SANHANES) [42] where it was found that vitamin A deficiency in children under five decreased from 63.6% in the NFCS of 2005 [46] to 43.6% in 2012, a 20 % decrease. Key contributors to vitamin A intake in the 2018 PDIS were in descending order organ meats (although the per capita intake of liver was low as mentioned above), maize porridge, vitamin A-rich vegetables and medium-fat margarine. Mitsopoulou et al. [43] reported that processed cereals and milk were the most important sources for Greek children, with vegetables not featuring under the top 10 sources. The adequacy of vitamin A intake in the WC despite the much lower intake of maize porridge and similar intakes of bread, vitamin A-rich fruits and vegetables, liver and eggs is thus interesting. One explanation could be the significantly higher per capita intake of the ‘Other starches’ group in the WC that included sweet potatoes (orange flesh sweet potatoes provide 2182 mcg/100 g vitamin A [27]) along with potatoes in our adapted FAO–FANTA grouping [30]. Based on the significantly higher dairy intake in the WC, it is possible that dairy products fortified with vitamin A, such as some milk powders, may have played a role.

Low fruit and vegetable intake is a widespread problem in South Africa on national, household and individual levels in both children and adults [31,39]. Estimation of fruit and vegetable intake (combined) from the 1999 NFCS data showed that 1- to 3-year-olds consumed 180 g, 4- to 6-year-olds 206 g and 7- to 9-year-olds 237 g. This was well below the recommendation made by Naude [31] for South Africans, namely that parents should aim for at least 320 g of vegetables and fruit every day (four servings of 80 g) for older children in the pre-school age group and for school children and adults at least 400 g of vegetables and fruit every day (five servings of 80 g). These amounts are believed to provide sufficient micronutrients, particularly vitamin A, vitamin C, folate, vitamin E, potassium and fiber in the diet [47]. It is thus a great concern that the 2018 PDIS results show that the combination of fruit (including fruit juices) and vegetable intakes have in fact decreased to approximately 110 g in all three age groups. A trend for vegetable intake to have been higher in GTG and fruit intake higher in WC is evident. 

This low level of fruit and vegetable intake combined with the fact that vitamins C and E are not part of the fortification mix, may be linked to the findings that percentage intake below the EAR was more common for these two nutrients than for the fortification micronutrients. Median intakes were above the EAR for all age groups, but percentage vitamin C below the EAR was 25% and for vitamin E 19% in the 6- to <10-year-olds. Vitamin C intake, which is essential for normal collagen production, energy metabolism, immunity and antioxidant activities [48] was significantly lower in GTG than the WC in all age groups, which could be linked to the lower fruit (including fruit juice) intake in GTG. Fruit juice, including vitamin C-rich juice, and vitamin C-rich vegetables contributed 56% to total vitamin C intake in the total 2018 PDIS sample. 

As can be expected, PUFA oils and medium fat margarine contributed most to total vitamin E intake (30.4%), salty snacks contributed 8.9% and vitamin C-rich vegetables and eggs between 5–6% each. Risk of vitamin E or vitamin C deficiency was not reported for rural Zambian 4- to 8-year-olds [33], Senegalese 5- to 17-year-olds [34] or pre-school Mexican children [49]. Bailey et al. [50] claim that dietary vitamin E intakes in U.S. children is generally below recommended values, except for those children taking supplements. Low vitamin E intake was also very prominent in children in the HHNHS study in Greece Salty snacks, processed cereals and olives were the top sources of Greek children [40]. Vitamin E is an essential fat-soluble antioxidant [51] and Mitsopoulou et al. [43] contend that deficiency is more common among children due to their low body reserves and increased needs as a result of intense growth and development.

Two nutrients that are not included in the fortification mix that are essential for normal growth, development and health in children are calcium and vitamin D [51,52]. It is thus with great concern that we note that there seems to have been little improvement in the intake of these nutrients since 1999 [2]. Calcium intake was further significantly lower in GTG than the WC in the two older age groups. Although dairy was the top contributor to calcium intake, the per capita intake was generally low and decreased from the youngest to the oldest age group. Swanepoel et al. [35] reported that approximately 75% of 12- and 18-month-old children from an urban low socio-economic area in the North West province of South Africa had low calcium intake. Inadequate calcium intakes were also evident in the urban Senegalese 5- to 17-year-olds [34] and rural 4- to 8-year-old Zambian children [33], but not in Mexican pre-school children [49]. Pettifor [53] indicated that dietary calcium intakes of children in developing countries are frequently one third to one half of those in developed countries.

The consequences of a low calcium (dairy) intake have been described by numerous researchers. Very low calcium (less than 200 mg/day) diets greatly increases the risk of rickets in children and osteoporosis in adult life [53]. A study of children [54] who avoided milk were found to have a smaller stature, lower total-body bone mineral content and lower z scores for areal bone mineral density at the lumbar spine, femoral neck, hip trochanter and ultradistal radius, than did control children of the same age from the same community. Moreover, a meta-analysis of 21 randomized control trials [55] found that increased dietary calcium/dairy products, with and without vitamin D, significantly increased total body and lumbar spine bone mineral content in children with low baseline intakes. These results emphasize the importance of children meeting the food-based dietary guidelines (FBDG) (pediatric or those for 7 years and older) [56,57] of consuming dairy, especially in light of the fact that no mandatory fortification of foods with calcium is in place in South Africa.

The median vitamin D intake in the PDIS was below the EAR (10mcg) in all three age groups and 98–100% of children in each age group had an intake below the EAR. This is in line with intakes below the EAR of 99–100% reported for Greek 1- to 13-year-olds [43]. Moreover, Jiménez-Aguilar et al. [49] mention that vitamin D deficiency in children may be an emerging concern in Latin America, although it did not emerge as a problem in urban Senegalese [34] and rural Zambian children [33]. 

Vitamin D occurs naturally in a limited number of foods that are typically scarcely consumed, making it difficult to achieve the recommended dietary intake [43]. Best food sources include fatty fish, canned pilchards, sardines, tuna canned in oil, margarines if fortified, eggs, beef liver and fortified cereals [27,52]. To meet the RDA of 15 mcg a child would need to eat 120 g fatty fish such as fresh salmon or approximately 200 g canned pilchards/sardines or 300 g of tuna canned in oil or 300 g margarine or 200 g eggs or 900 g beef liver or 500 g fortified cereals, or an appropriate combination of these foods each day. Per capita intake of canned pilchards/sardines/tuna in the 2018 PDIS was less than 15 g, of eggs approximately 10 g, of fats and oils (thus not just margarines) between 8 g and 19 g (from youngest to oldest group), of fortified cereals less than 10 g and liver less than 2 g. Vitamin D content for all these foods is available in the SAMRC Food Data base [27], reducing the likelihood that we underestimated vitamin D intake in the 2018 PDIS. 

Of note is that inadequate vitamin D intake does not necessarily result in vitamin D deficiency, as sun exposure is recognized as an important source of vitamin D in countries where foods are not fortified with vitamin D [54]. There is a paucity of research on the actual vitamin D status of South Africans [58]. One of the few studies in children was conducted in Johannesburg, GTG, on a sub-cohort of the longitudinal Birth to Twenty study. They reported that 7% of 10-year-old children had a biochemical vitamin D deficiency and indicated at the time that they would not recommend vitamin D fortification or supplementation [59]. 

According to Norval et al. {58] earlier studies conducted in the same area in the late 1970s found no biochemical vitamin D deficiency in 7- to 12-year-old children. However, the Ultraviolet Index and number of hours of sunshine per day in the winter and summer are less favourable for vitamin D production in Cape Town (WC) than Pretoria (70 km north of Johannesburg) (GTG) [58]. The possibility that children in Cape Town may be worse off in terms of biochemical vitamin D status is illustrated by the results of a comparative survey of adolescents (*n* = 162) with and without alcohol abuse disorders living in the Cape Metropole [60]. Only 11% of those with alcohol use disorders and 29.6% of those without such disorders were found to be vitamin D sufficient (serum-25-Hydroxy vitamin D: ≥30 ng/mL), with dietary vitamin D intake being well below the EAR of 10mcg at 2.5 mcg and 3 mcg respectively. 

A limitation of this research is that self- or by proxy reporting of food intake data using 24-h recall introduces bias, including misreporting because of respondent memory lapses (error of omission) or because of reporting foods that were not consumed (error of commission), as well as incorrect estimation of portion size consumed, to the dietary intake data [57]. However, as recommended by Gibson [61], the multiple pass method was used to reduce error of omission and life size sketches of household utensils and foods, as well as generic three dimensional food models were used to aid portion size estimation. Furthermore, children (7 years and older) were interviewed in the presence of the parent or primary care giver and if child had been at a day care center the previous day, the center was visited by the fieldworker and the meals and portion sizes were determined for the 24 h in question. Although a strong limitation of this study is that the sample was not representative of the all nine provinces in the country, a key is that it is representative of the three age groups investigated in the two provinces. A further strength is that we completed two additional 24-h recalls in a 9% subsample to apply the National Cancer Institute (NCI) method [17,18] to obtain results that are more representative of usual intake.

## 5. Conclusions

To conclude, our results show that fortification of maize and bread flour most probably made a considerable contribution to improvement of the intake of nutrients included in the fortification mix, namely iron, zinc, vitamin A, thiamine, riboflavin, niacin, vitamin B6 and folate in the total PDIS sample representing the WC and GTG. However, folate intake still needs attention, especially in the WC. Maize porridge was most probably the most important fortification vehicle, especially in GTG, with bread making a limited contribution in both provinces. Despite the significantly lower intake of maize porridge by children in the WC than in GTG, the only material difference in intake was evident for folate. Based on this scenario it seems possible for children to obtain the necessary iron, zinc, thiamine, riboflavin, niacin and vitamin B6 from the diet without eating the amount of maize porridge and bread eaten by children in GTG. Intake of flesh foods, including chicken liver, as well as dairy and eggs, may make a material contribution in this regard.

Nutrients that are not included in the fortification mix that remain a very serious concern are calcium and vitamin D, with dairy intake being especially low in GTG and intake of vitamin D sources being very limited in both provinces. Bearing in mind the high prevalence of inadequate vitamin D intake found in this study, as well as recommendations to avoid sun exposure for skin cancer prevention, it is essential to gain more insights into the biochemical vitamin D status of children in the different areas in the country to consider the need for mandatory fortification of foods with vitamin D, or more targeted, area-specific interventions. A further concern is the low overall intake of fruits and vegetables in the two provinces, which provide fiber and many other biologically active compounds important for health over and above vitamins A and C. It is evident that the 6– to <10-year-olds may be most vulnerable to dietary risk for micronutrient deficiencies. 

We recommend the following: in the short term, promotion of intake of fruit, vegetables, especially also dark green leafy vegetables, as well as other folate sources such as liver, especially chicken liver, should be implemented as a matter of urgency in the WC and GTG. Guidelines for safe sun exposure for vitamin D production should also be formulated and disseminated. In the medium and longer term, mandatory fortification of additional food items with folate and appropriate food vehicles with vitamin D and calcium should be investigated. 

These recommendations need to be considered in the context that the findings may not be representative of the other seven provinces in South Africa that are less urbanized and economically active. 

## Figures and Tables

**Table 1:** Sociodemographic and other characteristics of parents and 1- to <10-year-old children in the two provinces studied.

	Gauteng N = 733 % (95% CI)	Western Cape N = 593 % (95% CI)	Rao–Scott Chi-Sq Values	All N = 1326 % (95% CI)
Primary caregiver				
Mother	70.1 (65.6–74.6)	71.0 (64.7–77.2)	0.045	70.4 (66.8–74.0)
Father	6.6 (3.4–9.7)	1.8 (0.2–3.3)		5.0 (2.8–7.1)
Grandparent	16.7 (12.9–20.4)	21.0 (15.5–26.4)		18.1 (15.0–21.2)
Other (e.g., sibling, aunt)	6.7 (4.0–9.5)	6.3 (2.1–10.4)		6.6 (4.3–8.8)
Age in years				
1- to <3-years	26.3 (22.1–30.6)	25.3 (19.4–31.2)	0.923	26.0 (22.6–29.4)
3- to <6-years	35.4 (31.0–39.8)	35.1 (30.7–39.5)		35.3 (32.1–38.5)
6- to <10-years	38.3 (34.1–42.4)	39.6 (33.1–46.1)		38.7 (35.2–42.2)
Gender				
Male	50.2 (45.5–54.9)	47.5 (43.1–51.9)	0.391	49.3 (45.9–52.7)
Female	49.8 (45.1–54.5)	52.5 (48.1–56.9)		50.7 (47.3–54.1)
Head of household				
Father	40.2 (33.8–46.6)	38.8 (34.6–43.0)	0.132	39.7 (35.3–44.1)
Mother	16.8 (13.8–19.9)	10.8 (7.0–14.5)		14.8 (12.5–17.2)
Grandmother	21.9 (15.5–28.3)	28.3 (21.8–34.9)		24.0 (19.3–28.8)
Grandfather	11.7 (8.3–15.1)	14.0 (10.0–18.0)		12.5 (9.9–15.0)
Other (e.g., aunt, uncle)	9.4 (5.7–13.1)	8.1 (4.9–11.4)		9.0 (6.3–11.7)
Marital status of mother				
Unmarried	41.1 (34.9–47.2)	34.8 (28.4–41.1)	<0.001	39.0 (34.4–43.5)
Married	24.9 (20.5–29.4)	41.3 (33.3–49.2)		30.4 (26.4–34.3)
Divorced/ widowed	4.8 (2.5–7.0)	2.4 (0.7–4.2)		4.0 (2.4–5.6)
Living together	27.8 (22.0–33.6)	20.8 (15.9–25.7)		25.5 (21.4–29.6)
Other	1.4 (0.2–2.6)	0.8 (0.0–1.8)		1.2 (0.3–2.1)
Mother’s highest education				
Not completing Gr. 12	51.2 (44.9–57.4)	57.7 (47.1–68.3)	0.183	53.3 (47.9–58.7)
Completion of Gr. 12	33.9 (28.4–39.4)	24.7 (17.6–31.8)		30.8 (26.5–35.2)
Qualification after Gr.12	12.2 (8.7–15.7)	15.6 (7.6–23.6)		13.3 (9.9–16.8)
Do not know	2.8 (1.4–4.1)	2.0 (0.5–3.5)		2.5 (1.5–3.5)
Father’s highest education				
Not completing Gr. 12	26.9 (22.0–31.7)	33.8 (29.0–38.5)	0.323	29.1 (25.6–32.7)
Completion of Gr. 12	32.6 (26.9–38.3)	30.4 (25.2–35.6)		31.9 (27.8–36.0)
Qualification after Gr.12	13.1 (9.4–16.9)	10.7 (5.7–15.7)		12.3 (9.4–15.3)
Do not know	27.4 (22.4–32.4)	25.2 (19.7–30.6)		26.7 (22.9–30.4)
Mother’s employment status				
Yes	22.4 (17.8–26.9)	38.4 (31.0–45.9)	<0.001	27.7 (23.9–31.5)
No	74.6 (69.6–79.6)	60.2 (53.0–67.5)		69.8 (65.8–73.9)
Don’t know/ not applicable	3.0 (1.3–4.7)	1.3 (0.3–2.4)		2.5 (1.3–3.6)
Father’s employment status				
Yes	64.8 (60.6–69.1)	65.3 (59.7–70.9)	0.953	65.0 (61.6–68.4)
No	21.4 (17.5–25.3)	20.5 (15.1–25.9)		21.1 (18.0–24.2)
Don’t know/ not applicable	13.8 (11.1–16.4)	14.1 (10.2–18.1)		13.9 (11.7–16.1)
Wealth index quintiles				
One	21.1 (14.6–27.6)	17.7 (10.7–24.7)	0.263	20.0 (15.1–24.8)
Two	17.8 (12.0–23.6)	24.3 (20.0–28.6)		20.0 (15.9–24.0)
Three	21.3 (17.0–25.7)	17.0 (12.6–21.4)		19.9 (16.7–23.1)
Four	21.5 (16.7–26.3)	17.5 (12.4–22.6)		20.2 (16.6–23.7)
Five	18.3 (11.6–25.0)	23.5 (14.5–32.5)		20.0 (14.7–25.3)
Ethnicity				
Black African	97.8 (96.0–99.6)	27.6 (12.9–42.3)	<0.001	74.5 (69.5–79.4)
Mixed ancestry	2.2 (0.3–4.0)	68.0 (53.7–82.4)		24.1 (19.2–28.9)
Other	0.0 (0.0–0.1)	4.4 (0.6–8.2)		1.5 (0.3–2.7)
Type of residence				
Rural	2.4 (0.7–4.1)	6.6 (1.6–11.5)	0.194	3.8 (1.9–5.7)
Urban formal	88.9 (82.3–95.4)	86.8 (79.1–94.5)		88.2 (83.2–93.2)
Urban informal	8.7 (2.7–14.7)	6.6 (1.7–11.5)		8.0 (3.7–12.3)
Mother’s BMI [26]				
Underweight/normal BMI = <18.5 and 18.5–24.9 kgm^2^	33.3 (28.0–38.5)	29.1 (23.6–34.5)	0.002	32.0 (28.0–35.9)
Overweight BMI = 25–29.9 kgm^2^	27.7 (23.6–31.8)	20.4 (16.5–24.3)		25.4 (22.4–28.5)
Obese BMI ≥ 30 kgm^2^	39.1 (35.8–42.3)	50.6 (43.0–58.1)		42.6 (39.4–45.8)
Hunger scale [25]				
Total score = 0: No risk	57.9 (49.5–66.3)	48.8 (38.9–58.7)	0.1483	54.9 (48.5–61.3)
1–4: At risk of hunger	22.1 (17.2–27.0)	28.9 (23.0–34.9)		24.4 (20.6–28.2)
5–8: Food shortage in house	20.0 (14.8–25.1)	22.3 (16.5–28.0)		20.7 (16.8–24.6)

CI = 95th Confidence Interval, Gr. = grade, BMI = body mass index.

**Table 2:** Mean (CI), median (IQR) and percentage intake below the EAR for mineral intake (adjusted) of children aged 1 –<10-years by province and age.

Age in Years		1- to <3-Years			3- to <6-Years			6- to <10-Years		
Province		GTG	WC	All	GTG	WC	All	GTG	WC	All
N		185	148	333	282	232	514	266	213	479
Calcium (mg/day) EAR 1–3 yrs = 500 mg; 4–8 yrs = 800mg; 9yr-<10 yrs = 1100 mg (Lambda = 0.24)	Mean (CI)	370.9 (265.5–476.3)	532.8 (351.9–713.6)	423.3 (312.4–534.1)	296.3 *** (277.2–315.4)	461.4 (441.4–481.4)	350.9 (335.1–366.7)	330.3 * (283.9–376.6)	393.5 (370.1–417.0)	351.8 (318.5–385.1)
	Median (IQR)	334.5 (232.3–469.7)	486.9 (349.7–669.9)	378.0 (259.0–538.4)	287.4 (236.8–346.3)	449.5 (378.2–532.4)	329.2 (259.6–421.5)	311.6 (238.5–401.8)	373.6 (286.9–476.6)	331.3 (251.6–428.9)
	% < EAR	78.9 (58.0–100.0)	52.1 (22.0–82.2)	70.2 (51.1–89.3)	99.5 (96.0–100.0)	85.5 (81.1–89.8)	94.8 (91.5–98.2)	99.6 (98.8–100.0)	98.9 (97.2–100.0)	99.4 (98.4–100.0)
Phosphorus mg/day EAR 1–3 yrs = 380 mg; 4–8 yrs =405 mg; 9-<10 yrs = 1055 mg (lambda = 0.33)	Mean (CI)	550.6 (478.9–622.2)	627.8 (515.1–740.5)	575.5 (506.9–644.2)	594.5 (537.5–651.5)	624.4 (604.0–644.7)	604.4 (567.8–640.9)	695.4 (643.8–747.1)	700.6 (630.6–770.5)	697.2 (648.3–746.1)
	Median (IQR)	530.1 (422.3–530.1)	604.8 (488.6–745.8)	553.5 (440.4–687.9)	584.2 (498.4–678.7)	612.9 (526.8–709.6)	593.4 (507.2–689.1)	677.4 (564.5–807.8)	684.6 (567.0–814.3)	680.0 (565.4–810.0)
	% < EAR	16.3 (9.9–22.7)	8.5 (2.2–14.9)	13.8 (10.4–17.3))	5.2 (0.0–14.2)	3.4 (0.0–13.5)	4.6 (0.0–13.9)	16.8 (10.2–23.4)	24.1 (14.9–33.3)	19.3 (17.0–21.6)
Iron (mg/day) EAR 1–3 yrs = 3.0mg; 4–8 yrs = 4.1; 9-<10 = 5.9 mg (Lambda = 0.28)	Mean (CI)	7.8 (6.2–9.3)	7.7 (5.4–10.1)	7.8 (6.4–9.1)	8.9 (8.4–9.5)	8.8 (8.6–9.0)	8.9 (8.6–9.2)	11.0 ** (10.7–11.2)	9.9 (9.4–10.3)	10.6 (10.3–10.9)
	Median (IQR)	7.4 (5.8–9.3)	7.3 (5.7–9.3)	7.3 (5.7–9.3)	8.8 (7.7–10.0)	8.7 (7.6–9.8)	8.9 (7.7–10.0)	10.7 (9.1–12.5)	9.7 (8.2–11.3)	10.3 (8.7–12.2)
	% < EAR	1.0 (0.0–2.5)	1.1 (0.0–4.7)	1.0 (0.0–3.2)	0.02 (0.0–0.1)	0.0 (0.0–0.2)	0.01 (0.0–0.1)	0.1 (0.0–0.5)	0.5 (0.0–1.3)	0.3 (0.0–0.8)
Zinc (mg/day) EARs: 1–3 yrs = 2.2 mg 4–8 yrs = 4.0 mg 9–10 yrs = 7.0 mg (Lambda = 0.23)	Mean (CI)	6.4 (5.2–7.7)	6.6 (4.5–8.7)	6.5 (5.4–7.6)	7.4 (6.8–7.9)	7.1 (6.3–8.0)	7.3 (6.9–7.7)	8.8 ** (8.4–9.2)	7.7 (7.4–8.1)	8.5 (8.1–8.8)
	Median (IQR)	6.2 (5.0–7.6)	6.3 (5.1–7.8)	6.2 (5.0–7.7)	7.2 (6.2–8.40	7.0 (6.0–8.1)	7.1 (6.1–8.3)	8.6 (7.4–10.1)	7.6 (6.4–8.9)	8.3 (7.0–9.7)
	% < EAR	0.2 (0.0–0.5)	0.1 (0.0–0.8)	0.1 (0.0–0.5)	0.4 (0.0–1.1)	0.6 (0.0–2.7)	0.5 (0.0–1.6)	2.9 (1.5–4.3)	8.7 (2.8–14.6)	4.9 (2.7–7.0)

GTG = Gauteng; WC = Western Cape; EAR = Estimated Average Requirement; CI = 95th Confidence Interval; IQR = Inter quartile range, Independent *t*-tests showed significant differences between provinces; * *p* < 0.05, ** *p* < 0.01, *** *p* < 0.001. A Lambda value <0.15 reflects a larger mean bias as a result of sensitivity to the transformation applied.

**Table 3:** Mean (CI), median (IQR) and percentage intake below the EAR for vitamins A, D, E and C (adjusted) of children aged 1 to <10-years by province and age.

Age in Years		1 to <3 Years			3 to <6 Years			6 to <10 Years		
Province		GTG	WC	All	GTG	WC	All	GTG	WC	All
N		185	148	333	282	232	514	266	213	479
Vitamin A (ug/day) EARs 1–3 yrs = 210 ug 4–8 yrs = 275 ug 9 to <10 yrs = 445 ug (Lambda = 0)	Mean (CI)	570.5 (343.8–797.1)	582.1 (479.8–684.4)	574.2 (409.1–739.4)	641.5 (577.6–705.4)	537.2 (436.8–637.6)	607.0 (549.4–664.6)	633.7 (421.7–845.7)	604.4 (557.0–651.8)	623.8 (472.9–774.6)
	Median (IQR)	525.8 (403.3–687.5)	535.6 (411.7–704.1)	529.5 (405.6–692.7)	617.2 (508.8–746.7)	515.7 (428.4–624.4)	580.5 (476.2–710.0)	559.1 (402.3–780.7)	533.6 (383.2–744.8)	550.3 (395.3–768.8)
	% < EAR	1.3 (0.0–3.7)	1.0 (0.0–5.2)	1.2 (0.0–4.1)	0.2 (0.0–3.2)	0.8 (0.0–6.5)	0.4 (0.0–4.2)	10.5 (5.8–15.2)	14.8 (7.1–22.4)	12.0 (6.9–17.0)
Vitamin D (ug/day) EAR =10 ug 1- < 10 yrs (Lambda = 0.26)	Mean (CI)	2.3 (1.5–3.0)	4.0 (2.3–5.7)	2.8 (2.0–3.6)	2.3 (1.9–2.6)	2.6 (2.4–2.8)	2.4 (2.1–2.7)	3.0 (2.8–3.2)	3.8 (3.0–4.7)	3.3 (2.9–3.6)
	Median (IQR)	1.8 (1.0–3.0)	3.3 (2.0–5.3)	2.2 (1.2–3.8)	2.2 (2.0–2.7)	2.5 (2.3–3.0)	2.3 (2.1–2.8)	2.6 (1.8–3.8)	3.4 (2.3–4.8)	2.9 (1.9–4.2)
	% < EAR	99.4 (98.4–100.0)	95.6 (90.7–100.0)	98.2 (96.9–99.4)	100.0 (100.0–100.0)	100.0 (100.0–100.0)	100.0 (100.0–100.0)	99.6 (98.6–100.0)	98.6 (97.4–99.8)	99.3 (98.3–100.0)
Vitamin E (mg/day) EARs 1–3 yrs = 5 mg 4–8 yrs = 6 mg; 9- < 10yrs = 9.0 mg (Lambda = 0.16)	Mean (CI)	7.9 (7.6–8.3)	8.3 (6.7–10.0)	8.1 (7.4–8.8)	8.0 (7.0–8.9)	8.5 (8.3–8.7)	8.2 (7.6–8.7)	11.3 (10.4–12.1)	10.7 (9.6–11.7)	11.1 (10.1–12.0)
	Median (IQR)	7.3 (5.5–9.7)	7.7 (5.8–10.3)	7.3 (5.6–9.9)	7.3 (5.4–9.9)	7.8 (5.8–10.4)	7.5 (5.5–10.1)	10.3 (7.5–13.9)	9.7 (7.0–13.2)	10.1 (7.3–13.7)
	% <EAR	19.2 (10.0–28.4)	16.1 (2.2–30.0)	18.2 (7.9–28.4)	28.5 (8.2–48.8)	23.5 (13.4–33.7)	26.9 (10.2–43.5)	17.0 (6.7–27.4)	22.4 (11.2–33.5)	18.8 (8.5–29.2)
Vitamin C (mg/day) EARS 1–3 yrs =13 mg; 4–8 yrs =22 mg; 9 to <10 yrs =39 mg (Lambda = 0.29)	Mean (CI)	42.8 * (32.8–52.7)	57.8 (50.9–64.7)	47.6 (40.6–54.6)	32.4 ** (27.7–37.1)	53.7 (49.7–57.7)	39.4 (35.8–43.0)	35.5 ** (29.6–41.3)	55.9 (48.1–63.7)	42.4 (35.4–49.4)
	Median (IQR)	36.2 (22.0–56.0)	49.9 (31.9–75.5)	40.2 (24.5–62.8)	30.9 (23.7–39.6)	52.0 (41.6–63.9)	36.6 (26.8–49.1)	31.7 (21.2–45.5)	51.3 (35.8–70.6)	37.2 (24.5–54.7)
	% <EAR	9.2 (3.7–14.7)	3.6 (0.0–7.2)	7.4 (3.0–11.8)	13.2 (0.0–27.4)	0.6 (0.0–6.2)	9.0 (0.0–20.0)	32.1 (18.8–45.5)	11.9 (0.0–27.3)	25.3 (11.8–38.8)

GTG = Gauteng; WC = Western Cape; EAR = Estimated Average Requirement; CI = 95^th^ Confidence Interval; IQR = Inter quartile range, Independent *t*-tests showed significant differences between provinces; * *p* < 0.05, ** *p* < 0.01. A lambda value < 0.15 reflects a larger mean bias as a result of sensitivity to the transformation applied.

**Table 4:** Mean (CI), median (IQR) and percentage intake below the EAR for B vitamins (adjusted) of children aged 1 to <10 years by province and age.

Age in Years		1- to <3-Years			3- to <6-Years			6- to <10-Years		
Province		GTG	WC	All	GTG	WC	All	GTG	WC	All
N		185	148	333	282	232	514	266	213	479
Thiamine (mg/day) EARs 1–3 yrs = 0.4 mg; 4–8 yrs = 0.5 mg; 9 to <10 yrs = 0.7 mg (Lambda = 0.29)	Mean (CI)	1.0 * (0.9–1.1)	0.8 (0.7–1.0)	1.0 (0.9–1.0)	1.1 ** (1.0–1.1)	0.9 (0.9–1.0)	1.0 (1.0–1.1)	1.2 ** (1.1–1.3)	1.1 (1.0–1.1)	1.2 (1.1–1.3)
	Median (IQR)	1.0 (0.8–1.2)	0.8 (0.6–1.0)	0.9 (0.7–1.2)	1.1 (0.9–1.3)	0.9 (0.8–1.1)	1.0 (0.8–1.2)	1.2 (1.0–1.4)	1.0 (0.8–1.2)	1.1 (0.9–1.4)
	% < EAR	0.6 (0.0–2.6)	3.6 (0.0–8.9)	1.6 (0.0–4.8)	0.3 (0.0–1.9)	1.5 (0.0–4.3)	0.7 (0.0–2.7)	0.6 (0.0–1.6)	2.8 (0.0–6.2)	1.3 (0.0–3.1)
Niacin (mgNE/day) EARs 1–3 yrs = 5.0; 4–8 yrs 6.0 mgNE, 9 to <10 yrs = 9.0 mgNE (Lambda = 0.41)	Mean (CI)	11.3 (10.2–12.4)	11.9 (9.3–14.6)	11.5 (10.6–12.4)	13.7 * (12.5–14.8)	15.2 (14.4–16.1)	14.2 (13.6–14.8)	17.4 (16.5–18.3)	17.0 (15.6–18.4)	17.2 (16.3–18.2)
	Median (IQR)	11.1 (9.6–12.8)	11.7 (10.2–13.6)	11.3 (9.8–13.0)	13.3 (11.0–16.0)	14.9 (12.4–17.7)	13.8 (11.4–16.6)	16.9 (13.9–20.3)	16.6 (13.6–19.9)	16.8 (13.8–20.2)
	% < EAR	0.1 (0.0–1.1)	0.0 (0.0–0.7)	0.05 (0.0–0.9)	0.4 (0.0–1.2)	0.1 (0.0–0.6)	0.3 (0.0–1.0)	0.4 (0.0–0.8)	0.7 (0.2–1.2)	0.5 (0.2–0.8)
Riboflavin (mg/day) EARs 1–3 yrs = 0.4 mg; 4–8 yrs = 0.5 mg; 9 to <10 yrs = 0.8 mg (Lambda = 0.22)	Mean (CI)	0.8 (0.6–1.1)	1.1 (0.9–1.3)	0.9 (0.7–1.1)	0.8 ** (0.7–0.9)	1.0 (1.0–1.1)	0.9 (0.8–1.0)	0.9 * (0.9–1.0)	1.1 (1.0–1.2)	1.0 (0.9–1.0)
	Median (IQR)	0.8 (0.6–1.0)	1.0 (0.8–1.4)	0.8 (0.6–1.1)	0.8 (0.7–1.0)	1.0 (0.9–1.2)	0.9 (0.7–1.1)	0.9 (0.7–1.1)	1.0 (0.8–1.3)	0.9 (0.7–1.2)
	% < EAR	7.4 (0.0–15.9)	1.8 (0.0–7.7)	6.0 (0.0–12.6)	3.3 (0.0–10.6)	0.3 (0.0–2.9)	2.3 (0.0–8.1)	12.5 (4.0–21.0)	9.5 (5.0–13.9)	11.4 (5.9–18.0)
Vitamin B_6_ (mg/day) EARs 1–3 yrs = 0.4 mg; 4–8 yrs 0.5 mg; 9 to <10 yrs = 0.8 mg (Lambda = 0.21)	Mean (CI)	1.4 (1.2–1.6)	1.3 (1.0–1.6)	1.4 (1.3–1.5)	1.9 * (1.8–2.0)	1.7 (1.6–1.9)	1.8 (1.8–1.9)	2.6 ** (2.5–2.7)	2.3 (2.2–2.4)	2.5 (2.4–2.6)
	Median (IQR)	1.4 (1.2–1.6)	1.2 (1.0–1.5)	1.3 (1.1–1.6)	1.8 (1.6–2.1)	1.7 (1.5–2.0)	1.8 (1.5–2.1)	2.5 (2.0–3.0)	2.2 (1.8–2.7)	2.4 (1.9–2.9)
	% < EAR	0.0 (0.0–0.0)	0.0 (0.0–0.0)	0.0 (0.0–0.0)	0.0 (0.0–0.0)	0.0 (0.0–0.0)	0.0 (0.0–0.0)	0.0 (0.0–0.1)	0.0 (0.0–0.3)	0.0 (0.0–0.1)
Vitamin B_12_ (ug/day) EARs 1–3 yrs = 0.7 ug; 4–8 yrs = 1.0 ug; 9 to <10 yrs =1.5 ug (Lambda = 0.13)	Mean (CI)	1.8 * (1.2–2.3)	3.0 (2.2–3.7)	2.2 (1.8–2.5)	2.7 ** (2.4–2.9)	3.4 (3.0–3.9)	2.9 (2.6–3.2)	4.0 (3.3–4.7)	4.8 (3.3–6.3)	4.3 (3.3–5.2)
	Median (IQR)	1.4 (0.8–2.3)	2.4 (1.5–3.9)	1.7 (1.0–2.8)	2.6 (2.3–3.1)	3.4 (2.9–3.9)	2.9 (2.4–3.4)	3.3 (2.1–5.1)	4.0 (2.5–6.2)	3.5 (2.2–5.5)
	% < EAR	18.7 (0.0–70.9)	5.0 (0.0–23.5)	14.3 (0.0–56.4)	0.0 (0.0–7.3)	0.0 (0.0–2.5)	0.0 (0.0–5.7)	6.2 (0.0–15.0)	4.0 (0.0–9.8)	5.4 (0.0–13.2)
Folate (ug/day) EARs 1–3 yrs= 120 ug; 4–8 yrs = 160 ug; 9 to <10 yrs =250 ug (Lambda = 0.1)	Mean (CI)	252.0* (207.6–296.3)	169.8 (106.4–233.2)	225.4 (194.2–256.6)	281.6 *** (265.0–298.2)	195.7 (181.5–209.9)	253.2 (243.4–263.0)	313.5 *** (294.4–332.6)	221.1 (205.5–236.6)	282.1 (263.7–300.5)
	Median (IQR)	236.7 (185.5–302.2)	158.3 (123.5–205.1)	210.1 (157.7–274.5)	269.0 (215.0–332.6)	185.3 (149.0–231.7)	238.7 (185.4–305.9)	297.4 (238.6–370.7)	210.1 (166.9–262.3)	266.0 (205.7–340.3)
	% < EAR	3.3 (0.5–6.1)	22.6 (0.0–54.0)	9.6 (0.0–22.1)	3.9 (0.0–14.9)	23.3 (9.1–37.5)	10.3 (0.0–22.2)	6.9 (3.4–10.3)	32.3 (24.3–40.2)	15.5 (12.4–18.6)

GTG = Gauteng; WC = Western Cape; EAR = Estimated Average Requirement; CI = 95th Confidence Interval; IQR = Inter quartile range; yrs = years. Independent *t*-tests showed significant differences between provinces; * *p* < 0.05, ** *p* < 0.01, *** *p* < 0.001. A lambda value < 0.15 reflects a larger mean bias as a result of sensitivity to the transformation applied.

**Table 5:** Food items that contributed at least 5% to the total intake of calcium, iron and zinc of children aged 1 to <10 years (N = 1326).

Food Items- Calcium (Ca)	% Consumers	Average Ca per/Capita (mg)	Average Ca /Day (mg) of Consumers Only	% of Total Calcium of Total Consumers
Whole milk	47.6	88.0	185.0	23.4
Maize porridge	74.4	46.6	62.6	12.4
Canned pilchards/sardines	9.2	27.5	298.5	7.3
Maas/sour milk	10.9	25.7	235.0	6.8
BMS	4.0	20.2	505.2	5.4
Yoghurt	12.5	19.9	159.0	5.3
**Food Item-** **Iron (Fe)**	**% Consumers**	**Average Fe per Capita (mg)**	**Average Fe /Day** **(mg) of Consumers Only**	**% of Total Fe** **of Total Consumers**
Maize porridge	74.4	2.3	3.1	24.8
White bread	38.8	1.0	2.6	10.7
Brown bread	25.5	0.7	2.5	7.9
Cereal-high fiber	18.3	0.5	2.8	5.5
**Food Items-Zinc** **(Zn) Sources**	**% Consumers**	**Average Zn per Capita (mg)**	**Average Zn/Day (mg) of Consumers Only**	**% of Total Zn of Total Consumers**
Maize porridge	74.4	2.1	2.8	27.7
Brown bread	25.5	0.8	2.7	10.6
White bread	38.8	0.6	1.5	7.9
Beef	13.7	0.6	4.3	7.8
Chicken	45.4	0.5	1.1	6.6

Maas = a traditional sour drink made from maize; BMS = breast milk substitute. The average per capita consumption is the intake of the total sample of children of a particular nutrient from a particular food item divided by the total sample size. The average consumption per consumer is the intake of the total sample of children of a particular nutrient from a particular food item divided by the number of children who actually consumed the food item. The percentage intake of a particular nutrient from a particular food item is the amount of the nutrient provided by each individual food item for the total sample divided by the intake of the nutrient of the total group of children across all food items × 100.

**Table 6:** Food items which have contributed at least 5% of total vitamin A, D, E and C intakes of children aged 1 to <10 years (N = 1326).

Food Items-Vitamin A	% Consumers	Average Vit A/Day per Capita/Day (ug)	Average Vit A /Day of Consumers Only (ug)	% of Total Vit A Consumed	Food Items- Vitamin D	% Consumers	Average Vit D/Day per Capita (ug)	Average Vit D /Day of Consumers Only (ug)	% of Total Vit D Consumed
Organ meats	8.3	168.4	2029.7	25.4	Canned pilchards/ sardines	9.2	0.7	7.9	25.8
Maize porridge	74.4	153.5	206.3	23.2	Eggs	12.1	0.7	5.7	24.6
Vit A-rich veg	9.5	78.2	822.4	11.8	Med fat margarine	28.9	0.3	1.0	10.1
Med fat margarine	28.9	32.9	113.9	5.0	BMS	4.0	0.2	5.6	7.9
**Food Items-Vitamin E**	**% Consumers**	**Average Vit E/Day per Capita (mg)**	**Average Vit E /Day of Consumers Only (mg)**	**% of Total** **Vit E Consumed**	**Food Items- Vitamin C**	**% Consumers**	**Average Vit C/Day per Capita (mg)**	**Average Vit C/Day of Consumers Only (mg)**	**% of Total Vit C Consumed**
PUFA oil	14.9	1.6	10.5	17.2	Other fruit juice	6.6	8.1	122.2	18.8
Med fat marg	28.9	1.2	4.1	13.2	Vit C-rich fruit juice	9.5	7.0	73.4	16.2
Maize porridge	74.4	0.9	1.2	9.4	Potato/sw.potato	32.4	6.6	20.4	15.4
Salty snacks	49.3	0.8	1.6	8.9	Vit C veg group	27.9	4.8	17.0	11.0
Vit C veg group	27.9	0.5	1.9	5.8	Maize porridge	74.4	3.1	4.2	7.3
Eggs	12.1	0.5	4.0	5.4					
**Food Items- Vitamin B12**	**% Consumers**	**Average Vit B12/Day per Capita (ug)**	**Average Vit B12 /Day of Consumers Only (ug)**	**% of Total Vit B12 Consumed**	**Foods Times- Riboflavin/Vit B2**	**% Consumers**	**Average Vit B2/Day per Capita (mg)**	**Average Vit B2** **/Day of Consumers Only (mg)**	**% of Total** **Vit B2 Consumed**
Organ meat	8.3	1.2	14.0	31.6	Maize porridge	74.4	0.2	0.2	16.2
Canned pilchards/ sardines	9.2	1.1	12.2	30.5	Whole milk	47.6	0.1	0.2	11.8
Whole milk	47.6	0.3	0.6	7.8	Cereal-high fiber	18.3	0.1	0.4	8.2
Beef	13.7	0.3	1.9	7.0	Organ meats	8.3	0.1	0.8	7.3
					Cereal-low fiber	10.6	0.1	0.5	5.4
**Food Items- Folate**	**% Consumers**	**Average Folate/Day per Capita (ug)**	**Average Folate/Day of Consumers Only (ug)**	**% of Total Folate Consumed**	**Food Items-** **Thiamine (vit B1)**	**% Consumers**	**Average Vit B1/Day** **per Capita (mg)**	**Average Vit B1/Day of Consumers Only (mg)**	**% of Total** **Vit B1 Consumed**
Maize porridge	74.4	122.8	164.9	47.6	Maize porridge	74.4	0.4	0.5	38.1
Brown bread	29.5	23.0	78.1	8.9	White bread	38.8	0.1	0.2	7.8
White bread	38.8	22.1	56.9	8.6	Brown bread	29.5	0.1	0.3	7.7
Organ meats	8.3	18.3	220.8	7.1	Cereal-high fiber	18.3	0.1	0.3	5.7
**Food Items- Niacin**	**% Consumers**	**Average Niacin/Day per Capita (mg)**	**Average Niacin /Day of Consumers Only (mg)**	**% of Total Niacin Consumed**	**Food Items-Vitamin B6**	**% Consumers**	**Average Vit B6/Day** **per Capita (mg)**	**Average Vit B6/Day of Consumers Only (mg)**	**% of Total** **Vit B6 Consumed**
Maize porridge	74.4	2.9	3.9	19.7	White bread	38.8	0.5	1.2	24.3
Chicken	45.4	2.7	6.0	18.5	Maize porridge	74.4	0.5	0.6	22.9
White bread	38.8	1.7	4.3	11.1	Brown bread	29.5	0.4	1.3	19.3
Brown bread	29.5	1.5	4.9	9.8	Potato/sw.potato	32.4	0.1	0.3	5.3
Cereal-high fiber	18.3	0.8	4.2	5.2					
Potato/sw.potato	32.4	0.7	2.3	5.0					

Vit = vitamin; sw. = sweet; salty snacks mainly crisps, BMS = breast milk substitute. The average per capita consumption is the intake of the total sample of children of a particular nutrient from a particular food item divided by the total sample size. The average consumption per consumer is the intake of the total sample of children of a particular nutrient from a particular food item divided by the number of children who actually consumed the food item. The percentage intake of a particular nutrient from a particular food item is the amount of the nutrient provided by each individual food item for the total sample divided by the intake of the nutrient of the total group of children across all food items × 100.

**Table 7:** Mean (CI) per capita intake (g) of the adapted Food and Agriculture Organization–Food and Nutrition Technical Assistance Project (FAO–FANTA) [30] food groups of children aged 1 to <10 years by age and province.

Age in Years	1- to <3-Years-Old			3- to <6-Years-Old			6- to <10-Years-Old		
	Mean Grams (CI)			Mean Grams (CI)			Mean Grams (CI)		
	GTG N = 185	WC N = 148	All N = 333	GTG N = 282	WC N = 232	All N = 514	CTG N = 266	WC N = 213	All N = 479
Bread	15.2 (12.8–17.6)	17.7 (12.6–22.7)	16.0 (13.8–18.2)	37.9 (32.7–43.1)	40.3 (34.4–46.2)	38.7 (34.8–42.6)	68.0 (55.0–81.0)	64.5 (54.8–74.2)	66.8 (57.7–75.9)
Maize porridge	273.6 *** (237.1–310.1)	54.1 (26.1–82.1)	202.6 (173.6–231.5)	254.0 *** (225.9–282.1)	52.8 (33.1–72.5)	187.5 (165.0–10.8)	258.4 *** (227.9–288.9)	81.5 (53.1–109.8)	198.3 (174.3–222.3)
Fortified BF cereals	3.3 (1.7–5.0)	2.6 (0.4–4.8)	3.1 (1.8–4.4)	5.4 (3.1–7.7)	3.7 (2.0–5.5)	4.8 (3.2–6.5)	8.5 (4.9–12.2)	6.4 (3.8–9.0)	7.8 (5.3–10.3)
Other starchy foods	134.3 * (108.5–160.2)	184.1 (150.6–217.6)	150.4 (130.0–170.9)	155.3 ** (121.8–188.7)	238.9 (207.9–269.8)	182.9 (158.8–207.0)	158.3 ** (134.7–181.9)	219.7 (192.7–246.8)	179.2 (161.1–197.3)
Legumes and nuts	9.5 (3.5–15.4)	6.5 (0.1–12.9)	8.5 (4.0–13.0)	14.9 (8.3–21.5)	8.7 (2.5–14.9)	12.9 (8.0–17.7)	16.8 (9.6–23.9)	15.6 (8.7–22.5)	16.4 (11.2–21.5)
Dairy	127.5 ** (96.3–158.7)	250.5 (186.6–314.3)	167.3 (138.0–196.6)	102.7 ** (77.1–128.2)	182.9 (145.4–220.3)	129.2 (107.5–150.8)	100.1 * (82.2–117.9)	139.0 (112.0–166.1)	113.3 (98.3–128.3)
Cheese	0.2 (0.0–0.6)	0.9 (0.0–2.1)	0.4 (0.0–0.9)	1.0 ** (0.3–1.7)	3.8 (1.8–5.7)	1.9 (1.1–2.7)	3.3 (1.6–5.0)	4.0 (1.8–6.1)	3.5 (2.2–4.8)
Fats and oils	9.6 * (6.9–12.2)	5.8 (3.2–8.5)	8.4 (6.4–10.4)	13.8 (10.2–17.5)	11.2 (8.1–14.3)	13.0 (10.3–15.6)	20.3 * (17.5–23.0)	15.5 (12.7–18.2)	18.6 (16.6–20.7)
Eggs	5.2 * (1.7–8.6)	17.7 (7.4–28.1)	9.2 (5.2–13.3)	8.2 (4.6–11.7)	8.0 (4.2–11.8)	8.1 (5.5–10.8)	9.1 (4.4–13.7)	14.5 (8.1–20.9)	10.9 (7.2–14.6)
Liver	0.8 (0.0–1.8)	2.6 (0.0–6.2)	1.3 (0.0–2.7)	1.9 (0.5–3.2)	0.9 (0.0–2.3)	1.5 (0.5–2.5)	1.4 (0.2–2.7)	2.5 (0.0–5.2)	1.8 (0.6–3.0)
Pilchards/sardines/tuna	8.5 * (2.4–14.6)	1.3 (0.0–2.6)	6.2 (2.0–10.3)	10.2 (3.2–17.2)	3.9 (1.3–6.5)	8.1 (3.4–12.8)	13.7 (8.5–18.8)	9.6 (1.8–17.5)	12.3 (8.0–16.6)
Flesh foods (other)	41.5 ** (31.1–52.0)	66.1 (50.8–81.4)	49.5 (40.9–58.1)	52.4 *** (45.6–59.3)	98.2 (80.1–116.3)	67.6 (59.9–75.2)	67.8 ** (55.3–80.4)	102.9 (84.9–120.9)	79.7 (69.2–90.3)
Fruit and veg: Vit A rich	26.8 (14.4–39.3)	17.1 (9.6–24.7)	23.7 (15.1–32.3)	24.1 (16.1–32.1)	17.8 (10.9–24.7)	22.0 (16.3–27.7)	14.0 (8.6–19.4)	19.1 (12.0–26.3)	15.8 (11.5–20.0)
Fruit: Other	59.8 (40.2–79.3)	53.3 (23.9–82.7)	57.7 (41.5–73.8)	53.1 (33.7–72.6)	77.8 (50.5–105.1)	61.3 (45.6–77.0)	52.0 (33.5–70.4)	85.6 (55.4–115.9)	63.4 (47.4–79.4)
Veg: Other ^1^	20.9 (15.6–26.3)	14.7 (6.1–23.4)	18.9 (14.3–23.5)	31.7 ** (23.5–39.9)	16.7 (8.6–24.9)	26.7 (20.8–32.8)	44.4 *** (35.0–53.8)	18.1 (10.2–26.0)	35.5 (28.7–42.2)
All other foods	192.8 ** (150.0–235.7)	320.7 (250.8–390.6)	234.2 (196.0–272.4)	187.1 (165.1–209.1)	196.1 (150.8–241.4)	190.1 (169.4–210.8)	293.2 (255.5–330.9)	278.6 (218.5–338.8)	288.3 (256.6–319.9)

BF: breakfast; veg: vegetables; CI: 95% Confidence Interval. ^1^ Excluding potatoes, sweet potatoes (included in ‘Other starchy foods’) and olives and avocadoes (included in ‘Fats and Oils’); Independent *t*-tests showed significant differences between provinces; * *p* < 0.05, ** *p* < 0.01, *** *p* < 0.0001.

**Table 8:** Multivariate logistic regression analyses for identification of socio-demographic predictors of bread and maize porridge intake of children 1- to <10-years-old during the single 24-h recall period available for the total sample (adjusted for gender).

Sociodemographic and Other Variables	Children 1- to <10-Years Bread (Yes) N = 1326 (*n* = 864) OR (95% CI)	Children 1- to <10-Years Maize Porridge (Yes) N = 1326 (*n* = 890) OR (95% CI)
Age in years		
1 to <3 years	Ref	Ref
3 to <6 years	2.40 (1.68–3.43) ***	0.65 (0.41–1.05)
6 to <10 years	3.69 (2.47–5.50) ***	0.75 (0.46–1.23)
Gender		
Male	Ref	Ref
Female	0.93 (0.65–1.34)	0.95 (0.66–1.36)
Wealth index quintiles		
One/Two/Three		Ref
Four		0.74 (0.46–1.19)
Five		0.35 (0.21–0.57) ***
Ethnicity		
Black African	Ref	Ref
Mixed ancestry	1.09 (0.75–1.59)	0.20 (0.11–0.37) ***
Other	2.60 (1.05–6.45) *	0.50 (0.08–3.14)
Province		
Gauteng		6.31 (3.03–13.12) ***
Western Cape		Ref
Type of residence		
Formal urban	Ref	Ref
Informal urban	0.71 (0.48–1.06)	1.74 (0.98–3.10)
Rural	1.50 (1.03–2.16) *	1.68 (0.90–3.16)
Hunger scale		
Total score = 0: No risk		Ref
1–4: At risk of hunger		1.32 (0.76–2.29)
5–8: Food shortage in house		2.01 (1.16–3.49) *

OR = Odds ratio; * Odds ratio significant, *p* < 0.05; *** Odds ratio significant, *p* < 0.001. N-values reflect the total number of children, *n* represents the number of children in the risk group, estimates are adjusted using relevant weighting.

## Supplementary Materials

The following are available online at https://www.mdpi.com/1660-4601/17/16/5924/s1: Table S1: Loading of household possessions included in the wealth index for 5 quintiles title. Table S2: Hunger scale items. Table S3: NCI method used in the study. Table S4: Sociodemographic and other characteristics of the 1- to <10-year-old children for the different dietary recalls. Supplementary Figures S1–S3: Median intakes for the investigated micronutrients derived from the 1999 NFCS report [9] and 2018 PDIS data according to the NFCS age groups compared to the EAR for each nutrient.
